# Recombinant Oncolytic Viruses: Hexagonal Warriors in the Field of Solid Tumor Immunotherapy

**DOI:** 10.3390/cimb47110878

**Published:** 2025-10-23

**Authors:** Cong Zhang, Qian Sun

**Affiliations:** 1 National Clinical Research Center for Obstetrics and Gynecology, Department of Gynecological Oncology, Tongji Hospital, Tongji Medical College, Huazhong University of Science and Technology, Wuhan 430030, China; zhangconghust@163.com; 2 Cancer Biology Research Center (Key Laboratory of the Ministry of Education, Hubei Provincial Key Laboratory of Tumor Invasion and Metastasis), Tongji Hospital, Tongji Medical College, Huazhong University of Science and Technology, Wuhan 430030, China

**Keywords:** immunotherapy, antiviral immunity, neutralizing antibody, oncolytic adenovirus, herpes simplex virus

## Abstract

In the past decade, research on recombinant oncolytic viral agents in the treatment of solid tumors has evolved from the initial stage of simple genetic engineering to the current stage of multiple pipelines of parallel clinical application and combination therapy. Compared with T-VEC, the classical therapeutic agent that only expresses GM-CSF, which was approved in 2015, most new oncolytic virus designs include diverse gene constructs to reduce toxic effects, enhance multiple antitumor immunity, avoid immune clearance, or enhance tumor targeting. The single route of administration that activates the inflammatory tumor immune microenvironment by intratumoral injection is no longer sufficient to meet the treatment needs of refractory solid tumors. In this review, we illustrated the construction patterns of typical recombinant oncolytic viral agents and their latest clinical trial progress. Secondly, we summarized the underlying mechanisms of the combined application of antiviral and antitumor immunity in the field of solid tumor immunotherapy. Finally, we explored the feasibility of the intravenous application of oncolytic viruses and their future development directions. We believe that the diversified treatment design of oncolytic viruses will bring more surprises to the immunotherapy of refractory tumors.

## 1. Introduction

The resurgence of oncolytic viruses (OVs) as a potent immunotherapy modality has been bolstered by significant advancements in gene editing and virus production technologies. These breakthroughs have not only enhanced the immunomodulatory capabilities of OVs but also opened up new clinical transformation prospects. There are always some newly developed oncolytic viruses that emerge with impressive clinical trial results. OVs are listed as “the most promising immunotherapy direction” along with chimeric antigen receptor T-cell immunotherapy (CAR-T) and immune checkpoint inhibitors (ICIs).

In the first half of 2025, two groundbreaking papers were published in the *Nature* and *Cell* journals. In one case, VG161 has been shown to modulate the tumor immune microenvironment (TIME) and benefit patients with advanced refractory hepatocellular carcinoma (HCC). VG161 is a novel oncolytic virus modified based on herpes simplex virus 1 (HSV-1). It deletes the neurotoxic gene ICP34.5 of HSV-1 and carries the IL-12, IL-15, IL-15Rα, and PD-1/PD-L1 blocking fusion proteins [1]. Cytokines interleukin-12 (IL-12), IL-15, and IL-18 can induce the activation and differentiation of natural killer (NK) cells [2]. The PD-L1 blocking fusion protein can alleviate immunosuppression in the TIME. Because this recombinant oncolytic virus carries multiple antitumor immunomodulatory factors, it can activate both acquired and innate immunity. The proliferation of CD8^+^ T cells and NK cells in the tumor microenvironment of pancreatic cancer after VG161 injection has been detected by single-cell sequencing and mass spectrometry imaging techniques [3]. The results of its phase 1 clinical trial in 37 patients with advanced liver cancer have shown TIME remodeling efficacy and no dose-limiting toxicities. The most common side effect is a manageable fever. The objective response rate (ORR) of VG161 is 17.65%, and the OS of these patients is 9.4 months (NCT04806464) [4]. The combination of VG161 and paclitaxel chemotherapy performed in a breast cancer xenograft mouse model has demonstrated proinflammatory TME production and pulmonary metastasis reduction in a preclinical study [5]. Currently, participants in phase 2 clinical trials on the efficacy of VG161 combined with nivolumab in HCC are being recruited (NCT05223816). Another agent, NDV-GT, has similarly been shown to have a disease control rate of up to 90.0% in a small sample of patients with relapsed/refractory tumors [6]. NDV-GT is a recombinant Newcastle disease virus expressing the porcine α1,3 GT gene, which can trigger hyperacute rejection through intravenous injection. An in situ gene editing approach was performed using CRISPR/Cas9 technology [7]. The preclinical study of this engineered OV enrolled twenty patients with relapsed or refractory metastatic cancer and showed a disease control rate of 90.0% (Chinese Clinical Trial Registry of WHO, ChiCTR2000031980) [6]. NDV-GT represents an innovative topic of exploration to break through the bottleneck in the field of OV development by introducing xenogeneic antigens and intravenous injection. Initial basic and preclinical studies are conducted in models of liver cancer, but formal clinical trials are designed to target a broad spectrum of patients with solid tumors. Phase 2/3 clinical trials of this agent are still ongoing.

Reviewing the development of oncolytic viruses in the field of solid tumor immunotherapy, there are many typical agents that have completed clinical phase 2/3 clinical trials and have been approved for clinical use, and some are in clinical phase 3 trials, which are expected to become original agents that will soon enter clinical application. The first oncolytic virus, H101, was approved in China in 2005 for use in combination chemotherapy for patients with head and neck cancer or esophageal squamous cell carcinoma. Based on the results of the phase 3 randomized clinical trial of H101, the ORR of those receiving H101 plus cisplatin-based chemotherapy is 78.8% in a cohort of 160 patients [8]. The main adverse events (AEs) of the intratumoral injection of H101 are fever (45.7%), injection site pain (28.3%), and influenza-like symptoms (9.8%) [9]. Ten years later, another oncolytic virus, T-VEC, was fully approved for the treatment of unresectable stage IIIB-IV melanoma in the USA and Europe. Based on the results of the phase 3 clinical trial of T-VEC, the durable response rate (objective response lasting continuously for more than 6 months) is 16.3% in a cohort of 436 patients. The most common AEs are fatigue (50.3%), chills (48.6%), and pyrexia (42.8%) [10]. T-VEC remains the sole oncolytic virus therapy approved by the FDA. JX-594 has attracted much attention for its breakthrough efficacy in the intravenous treatment of HCC, but its phase 3 clinical trial results are not satisfactory. G47Δ has been conditionally approved for the treatment of malignant glioma in Japan since 2021. In addition to these agents, there are some OVs under phase 3 clinical trials that are promising for approval in clinical use, such as RP1, CG0070, OH2, Olvi-Vec, and Reolysin.

In this review, basic information (Table 1) and the latest clinical trial conclusions (Table 2) regarding typical oncolytic agents that have finished phase 2/3 clinical trials were summarized. Agents with ongoing phase 3 trials are listed in Table 3. We discussed the unique aspects of construct design and clinical advances in each of these recombinant oncolytic viruses in sequence. Besides discussing these recombinant viral designs, the rationale and limitations of immune regulation by oncolytic viruses must be reviewed in order to understand the evolution of viral engineering. We discuss the limitations of oncolytic viruses, focusing on the mechanisms of modulating antitumor immunity. We also conclude with a discussion of the latest findings in biomarkers associated with oncolytic viral response, briefly. Different tumor types are classified in order to draw a blueprint for the development of oncolytic viruses in the next decade.

## 2. Representative Agents of Recombinant Oncolytic Viruses

The representative recombinant oncolytic viruses discussed in this section are diverse in many aspects, such as their mechanisms of action, viral vectors, and routes of administration. In addition to their ability to lyse tumor cells, they may have different immunomodulatory mechanisms against different tumors. These viral vectors include HSV, adenovirus, polio–rhinovirus chimera, vaccinia virus, reovirus, and Newcastle disease virus. Intratumoral injection, intravenous injection, third spaces such as intrathoracic and abdominal cavity injection, and intravesical instillation can also be used. We will describe the rationale for each agent and the conclusion of their clinical trials in detail.

### 2.1. H101

H101 (Oncorine) is an engineered oncolytic adenovirus with deleted E1B-55kD and E3 regions, which selectively replicates in cancer cells and produces an oncolytic effect. However, the mechanisms of the E1B-deleted adenovirus responsible for selective replication have not been fully defined. One hypothesis is that H101 can selectively replicate in tumor cells with the dysfunction of the Rb-p53 pathway [11]. Nevertheless, given the clinical efficacy of H101 based on its phase 3 clinical trial, research to expand its indications in other tumor types and combination regimens is continuing. For instance, the intratumoral injection of H101 in the B16F10 melanoma xenograft mouse model can increase CD8^+^ T-cell infiltration in the TME, reduce M2-type macrophages, and induce vascular endothelial cell pyroptosis. This provides theoretical support for H101 combined with PD-L1 antibody treatment [12]. Similarly, H101 combined with anti-PD-1 therapy has also been explored in colorectal cancer (CRC) and advanced HCC [13]. The intratumoral injection of H101 can increase the infiltration of T cells and decrease the proportion of regulatory T cells (Treg cells) in the CT26 CRC mouse model [14]. Another clinical trial of seventeen refractory cervical cancer patients resulted in an ORR of 70.6% after H101 injection. KMT2D and ADAP1 gene mutations may be explored as biomarkers for evaluating the effectiveness of H101 treatment [15]. A combination of H101 injection with concurrent chemoradiotherapy in locally advanced cervical cancer patients has also been evaluated [16]. Furthermore, H101 injection can enhance the effect of irreversible electroporation treatment for pancreatic cancer patients by activating the JNK-MAPK pathway and inducing tumor cell apoptosis [17].

Regarding the agent injection methods of H101, in addition to intratumoral injection, intraperitoneal injection has also been shown to have significant efficacy in patients with malignant ascites [18]. Increased proportions of CLEC10A^+^ dendritic cells (DCs) and GZMK^+^CD8^+^ T cells have also been identified in patients with malignant ascites after H101 injection (NCT04771676) [19]. At present, there are still more than a dozen clinical trials registered on the website ClinicalTrials.gov. The majority of them are from Chinese research institutions.

### 2.2. T-VEC

Talimogene laherparepvec (T-VEC) is an engineered oncolytic virus based on HSV-1 with neurovirulence factor ICP34.5 and original ICP47 deleted and expressing granulocyte–macrophage colony-stimulating factor (GM-CSF). GM-CSF is a typical innate immune mediator regulating inflammatory responses and is approved for use in cancer immunotherapy. GM-CSF always served as an immune adjuvant that activated, boosted, and prolonged antitumor immunity [20]. However, further studies have confirmed that GM-CSF also has the effect of inhibiting antitumor immunity [21]. For instance, in intrahepatic cholangiocarcinoma tumors infiltrated by stromal cells, high-frequency suppressive tumor-associated macrophages (TAMs), and myeloid-derived suppressor cells (MDSCs), patients have better overall survival with decreased GM-CSF expression. The blockade of GM-CSF can demonstrate the repolarization of TAMs and MDSCs [22]. T-VEC can only be administered by intratumoral injection directly. Real-world data have shown that unresectable melanoma patients with stage III or IVM1a and tumor location amenable to agent injection have better objective responses and outcomes [23]. This also confirms that immunotherapy is a double-edged sword, and it is necessary to accurately control its indications and injection location to achieve effective therapeutic effects.

Challenges in the preclinical, clinical, and regulatory development of T-VEC have been summarized in a recent review published in January 2023 [24]. Here, we update and summarize data on key clinical trial results from 2023 to the present. The phase 2 randomized clinical trial that enrolled 150 patients with resectable stage IIIB to IVM1a melanoma has finished its 5-year follow-up. The estimated 5-year OS increased from 62.7% in the surgery arm to 77.3% in the neoadjuvant plus surgery arm. The 5-year recurrence-free survival (RFS), event-free survival (EFS), and distant metastasis-free survival (DMFS) were all improved with neoadjuvant T-VEC plus surgery vs. surgery alone (NCT02211131) [25]. Another phase 2 clinical trial enrolled 198 patients with unresectable stage IIIB to IV melanoma who received T-VEC plus ipilimumab or ipilimumab alone. The ORR improved from 16.0% in the ipilimumab arm to 35.7% in the combination arm. The estimated 5-year OS increased from 48.4% in the ipilimumab arm to 54.7% in the combination arm (NCT01740297) [26]. Unfortunately, the global phase 3 trial of T-VEC plus pembrolizumab, which enrolled 692 unresectable IIIB to IVM1c melanoma patients, was terminated. The results demonstrated no significant improvement in PFS or OS in the combined arm vs. pembrolizumab-alone arm (NCT02263508) [27]. Some clinical trials in other tumors have also shown initial results. A single-arm, phase 2 clinical trial with eighteen difficult-to-resect cutaneous basal cell carcinomas has shown an ORR of 55.6% after six cycles of T-VEC injection (NeoBCC trial) [28]. Patients with stage II–III triple-negative breast cancer who received T-VEC plus neoadjuvant chemotherapy were studied in another phase 2 trial (NCT02779855). In summary, the safety and efficacy of T-VEC in the treatment of advanced melanoma are beyond doubt. We can also see the promise of TVEC in the field of preoperative neoadjuvant therapy and combination therapy.

### 2.3. JX-594

JX-594, also known as Pexa-Vec (pexastimogene devacirepvec), is a poxvirus with GM-CSF insertion at the thymidine kinase (TK) gene locus, which replicates selectively in TK-highly expressing cells. Cancer-selective replication can be activated through epidermal growth factor receptor (EGFR)/Ras pathway signaling [29]. The polyclonal antibody against tumor antigens that mediates complement-dependent cytotoxicity (CDC) is induced after the intravenous delivery of JX-594 and destroys cancer cells [30].

The safety of JX-594 after intravenous infusion renewed hope in the field of oncolytic viruses in 2011 [31]. The intratumoral or intravenous injection of JX-594 has been confirmed to be safe and efficacious in patients with advanced HCC (NCT00629759 and NCT00554372) [32,33]. The presurgical intravenous delivery of JX-594 was performed in nine colorectal cancer liver metastases or melanoma. Severe side effects were neutropenia and lymphopenia [34]. A recent phase 2 clinical trial attempted the intratumoral injection of JX-594 in soft tissue sarcoma patients and sequential avelumab and cyclophosphamide therapy. Although this treatment can improve the immune-deficient tumor microenvironment, the short-term efficacy was not as expected through the intratumoral injection of JX-594 [35]. The results from another study examining intravenous JX-594 reached a similar conclusion [36]. The efficacy of intravenous JX-594 combined with low-dose cyclophosphamide was examined in advanced breast cancer patients [37]. Its initial clinical trials demonstrated safety but did not show sustained clinical benefit in subsequent studies, possibly due to the dual role of GM-CSF in stimulating and suppressing the immune response. Further studies are needed to determine whether the combination of JX-594 and immune checkpoint inhibitor agents has therapeutic effects. The combination of JX-594 with a PD-1 inhibitor was evaluated in early-stage and advanced-stage murine renal cell carcinoma (RCC) models. Although TME remodeling and safety were confirmed, efficacy was not significantly increased using JX-594 combined with a PD-1 inhibitor compared to using a CTLA-4 inhibitor combined with a PD-1 inhibitor [38]. A phase 3 clinical trial aimed to evaluate sequential treatment with JX-594 and sorafenib in 142 advanced HCC patients. Unfortunately, the combination therapy was even less effective than sorafenib alone [39]. A solution does not seem to have been found regarding the current situation of JX-594; neither a single agent nor a combination agent leads to a significant improvement in therapeutic efficacy. This phenomenon of high expectations and poor results is not uncommon in the field of oncolytic virus development. Therefore, the in-depth mechanism of the agent itself regulating tumor immunity is the basis for designing rational agent use regimens.

### 2.4. G47Δ

G47Δ, also known as Teserpaturev, is a third-generation oncolytic HSV-1 developed by a Japanese team. It was provisionally approved by the Japanese MHLW in June 2021 and is the world’s first oncolytic virus approved for the treatment of malignant glioma [40]. The construction of G47Δ deletes the ICP34.5 and ICP47 genes and inactivates the ICP6 gene, enabling it to replicate only in cancer cells and enhance the tumor immune response. The absence of ICP47 leads to a further attenuation of viral replication in normal cells but enhances the stimulation of antitumor immune responses. The hTERT promoter is used to restore ICP6 in a tumor-specific manner. This modification makes viral replication dependent on the activity of a cellular ribonucleic acid reductase that is normally highly expressed only in proliferating tumor cells [41]. G47Δ is based on G207 with the ICP47 gene removed.

A phase 2 clinical trial was conducted in 2015 [42]. Among the thirteen patients with recurrent/progressive glioblastoma despite receiving radiation and temozolomide therapies who participated in the phase 1/2 clinical trials using two doses of G47Δ intratumorally, the one-year survival rate reached 38.5% (UMIN-CTR Clinical Trial Registry UMIN000002661) [43]. When the administration of G47Δ was increased to six doses, the one-year survival rate reached 84.2% (UMIN-CTR Clinical Trial Registry UMIN000015995). The most common adverse reactions were fever (89.5%), vomiting (57.9%), nausea (52.6%), lymphocytopenia (47.4%), and leukopenia (31.6%). The grade ≥3 AE of lymphocyte count decrease occurred in five patients. Increased tumor-infiltrating CD4^+^/CD8^+^ lymphocytes and persistent low numbers of Foxp3^+^ cells were detected in the TME of patients who responded to treatment [44]. Although the results of the G47Δ study are very surprising, there are no updated phase 3 clinical results and no evidence from other research groups.

### 2.5. RP1

RP1, also known as vusolimogene oderparepvec, is an oncolytic virotherapy based on HSV-1. It expresses both the GALV-GP-R protein and GM-CSF. GALV-GP-R is a truncated and fusogenic form of the envelope glycoprotein of gibbon ape leukemia virus, which can enhance the oncolytic ability of HSV [45]. Phase 2 clinical data showed that oncolytic viral RP1 in combination with nivolumab exhibited deep and durable responses in anti-PD-1-failed melanoma patients (NCT03767348). Of 140 enrolled patients, the ORR was 32.9%. Overall survival rates at 1 and 2 years were 75.3% and 63.3%, respectively. The most common AEs were fatigue (32.9%), chills (32.1%), and pyrexia (30.7%). A total of 18 patients had grade ≥ 3 TRAEs [45,46]. Although the literature data on RP1 is very sparse, the combination of RP1 and nivolumab for the treatment of patients with advanced melanoma is expected to receive FDA approval in 2025.

### 2.6. CG0070

CG0070, also known as cretostimogene grenadenorepvec, is an engineered oncolytic adenovirus with deleted E1A and expressing GM-CSF, which selectively replicates in retinoblastoma (Rb) defective bladder transitional cell carcinoma (BTCC) cells and produces an oncolytic effect [47]. An E2F-1 promoter was inserted to interrupt Rb-E2F interaction and conditionally replicated in and lysed tumor cells.

Its first phase 1 clinical trial enrolled 35 patients, and the results were published in 2012. Intravesical infusions of CG0070 were performed, and grade 1–2 bladder toxicities were the most observed AEs [48]. A phase 2 multicenter trial enrolled 45 high-grade non-muscle-invasive bladder cancer (NMIBC) patients who had no response to bacillus Calmette–Guérin (BCG). The result showed an overall 47.0% CR rate at 6 months [49]. A phase 3 trial with a total of 112 high-risk NMIBC patients was conducted, and the latest results showed that the CR rate at any time after monotherapy was 74.5%. The estimated DOR at 12 months and 24 months was 63.7% and 58.7% (NCT04452591). The estimated primary completion year is 2027.

Although the efficacy of this agent as a single agent has been very satisfactory, its combination with ICIs is also being explored. A phase 1b clinical trial enrolled 21 patients with cT2-4aN0-1M0 muscle-invasive bladder cancer and was performed using CG0070 together with nivolumab. The pathologic complete response (CR) rate was 42.1%, and the 12-month RFS rate was 70.4% (NCT04610671) [50]. Furthermore, the CR rates at 12 months and 24 months were supervised in the phase 2 CORE-001 trial evaluating CG0070 plus pembrolizumab (NCT04387461). The intravesical injection and monotherapy effect of CG0070 are the most unique. It is believed that if the phase 3 clinical trial results continue to be excellent, this agent will be a highlight in the field of oncolytic virus therapy for bladder cancer.

### 2.7. OH2

OH2 was independently developed in China and obtained clinical approval from the NMPA in 2018, becoming the first oncolytic virus in the world to enter clinical research using HSV-2 as a vector. OH2 deleted the HSV-2 neurovirulence gene ICP34.5 and immune escape gene ICP47 and expressed GM-CSF [51]. The systemic immune responses induced by OH2 included boosting CCL5 production and activating CD8^+^ T cells and NK cell cytotoxicity [52]. Different administration routes of OH2 have been compared in rabbit tumor models. Transarterial viroembolization was more efficient compared to intratumor or intravenous injection [53]. Meanwhile, current clinical trials mainly focus on intratumoral injection.

The results of a phase 1a/1b trial in 44 patients with unresected stage III-IV melanoma have shown no grade 3/4 AEs after OH2 injection. The ORR was 37.0% to 58.3% in different subgroups (NCT04386967) [54]. A phase 3 clinical trial is being conducted and will be primarily completed in 2026 (NCT05868707). Other indications and combination regimens are also in early clinical trials. The effectiveness of OH2 against GBM has been discussed through single-cell RNA sequencing. The infiltration of macrophages, CD4^+^, and CD8^+^ T cells in the TME has been identified. A phase 1/2 clinical trial is ongoing for patients with recurrent central nervous system tumors (NCT05235074) [55]. Metastatic esophageal and rectal cancer patients can also benefit from OH2. Increased CD3^+^ T cells and CD8^+^ T cells and PD-L1 overexpression have been detected in the TME after OH2 injection [56]. The ORR was reported as 0% and 6.7% in the OH2 group and combination group (OH2 + PD-1 inhibitor) in a phase 1/2 trial that enrolled 26 advanced or metastatic sarcoma patients. However, the grade 3/4 AE rate was 15.4% [57]. The safety and combination of OH2 with PD-1 or PD-L1 inhibitors need further investigation.

### 2.8. PVSRIPO

PVSRIPO, also known as Lerapolturev, a novel viral immunotherapy based on the polio–rhinovirus chimera, has a unique target of CD155, the poliovirus receptor, which is widely expressed by tumor cells in most solid tumors. The antitumor mechanisms of VSRIPO include the following: (1) the direct infection and killing of CD155-expressing tumor cells and (2) the infection of intratumoral antigen-presenting cells, which activates innate and adaptive antitumor immune responses and releases inflammatory cascades leading to persistent systemic antitumor immunity [58]. Innate antiviral type I interferon (IFN) responses are activated after PVSRIPO infection. However, this does not conflict with viral replication in tumor cells [59]. The infiltration of DCs and T cells has also been confirmed in the TME [60]. Glioma-associated macrophages and microglia (GAMM) are engaged and participate in the activation of PVSRIPO-induced antitumor inflammation [61].

The earliest toxicity and efficacy trials were validated by intrathecal administration in rodent models of glioblastoma multiforme neoplastic meningitis [62]. A phase 1 trial of intratumorally administered PVSRIPO in twelve unresectable melanoma patients was performed; the ORR was 33.0% (NCT03712358) [63]. Another phase 1 trial of PVSRIPO in 61 recurrent malignant glioma patients showed a better survival rate at 24 months. The median OS for these patients was 12.5 months, but safety remains controversial, as 19.0% of patients had grade 3 or higher adverse events (NCT01491893) [64]. PVSRIPO has received orphan agent designation from the FDA for the treatment of advanced melanoma (stage IIB-IV) and recurrent glioblastoma. A randomized phase 2 trial of PVSRIPO alone or in combination with lomustine for patients with recurrent grade IV glioblastoma has been completed. The ORR was 5.4% in the PVSRIPO group and 7.4% in the PVSRIPO plus lomustine group. The median 5-year OS was 7.0 months in the PVSRIPO group and 7.1 months in the PVSRIPO plus lomustine group without significance (NCT02986178).

### 2.9. Olvi-Vec

Olvi-Vec, also named Olvimulogene nanivacirepvec, is a recombinant vaccinia virus formed by replacing the TK, hemagglutinin, and F145L genes with three expression cassettes encoding the β-galactosidase, β-glucuronidase, and RLuc-GFP fusion proteins, respectively [65]. The virus can selectively infect malignant cells and replicate in ovarian cancer cells and lung cancer cells [66]. A phase 1 clinical trial has been conducted on eighteen patients with malignant pleural effusion. The activation of CD8^+^ T cells, NK cells, DCs, and cytokines was identified after the intrapleural administration of Olivi-Vec (NCT01766739) [67]. A phase 2 clinical trial of Olvi-Vec was conducted and followed by platinum-based chemotherapy and bevacizumab in a cohort of twenty-seven platinum-resistant/refractory ovarian cancer patients. The ORR was 54.0%, and the median OS was 15.7 months in all patients (NCT02759588) [68]. A phase 3 clinical trial, with the same design as the phase 2 trial, is under recruitment for ovarian cancer patients (NCT05281471).

### 2.10. REOLYSIN

REOLYSIN, also known as Pelareorep, is a type 3 oncolytic reovirus with a dual mechanism of action. Specifically, the infection of normal cells with Pelareorep leads to the autophosphorylation of double-stranded RNA-activated protein kinase R (PKR), which inhibits viral protein synthesis. However, activated Ras signaling inhibits the autophosphorylation of PKR and allows for viral protein synthesis, which results in the specific lysis of Ras-signaling-activated tumor cells. At the same time, it can induce tumor cells to release virus-associated molecular patterns (PAMPs), activate DC and CTL, and upregulate the expression of PD-L1 in tumor cells [69]. Early phase 1/2 clinical trials have confirmed the safety of Pelareorep, but the treatment effect of Pelareorep combined with chemotherapy in patients with metastatic pancreatic cancer (MPA) does not improve PFS compared with chemotherapy alone (NCT01280058) [70]. More attempts have been made in a cohort of thirty-four MPA patients, aiming to verify the antitumor immune responses of Pelareorep combined with gemcitabine. The median OS was 10.2 months, and the upregulation of PD-L1 was detected (NCT00998322) [71]. Therefore, a further evaluation of Pelareorep and pembrolizumab is ongoing in clinical trials of MPA patients [72]. As for metastatic breast cancer (mBC), a phase 2 clinical trial that enrolled seventy-four patients was performed. No significant differences in PFS or response rate were shown between paclitaxel alone or Pelareorep combined with paclitaxel. Only a longer OS of 17.4 months was detected in the combination group (NCT01656538) [73]. Another phase 2 randomized study on forty-eight metastatic hormone receptor-positive breast cancer patients has been finished. The results show that the 16-week ORR and median PFS of Pelareorep combined with paclitaxel were 31.0% and 12.1 months. The addition of avelumab to triple therapy did not increase efficacy (NCT04215146) [74]. The agent is also in clinical trials in prostate cancer (NCT01619813), lung cancer (NCT00503295), head and neck cancer (NCT01166542), multiple myeloma (NCT00984464), and other cancers. It has the potential to become an oncolytic agent for a broad spectrum of solid tumors targeting Ras pathway activation.

## 3. The Safety and Immunomodulatory Mechanisms of Oncolytic Viruses

Reviewing the development history of the above representative oncolytic viral agents, we can see that many agents have attracted much attention in early clinical trials. Promising agents are advanced to phase 3 clinical trials because of their innovative design and high tumor reactivity and treatment efficiency. However, we have also seen many agents fail to meet the expected goals or produce serious adverse reactions, and their clinical development is terminated in phase 3 clinical trials. Only agents that have been successfully tested in phase 3 clinical trials or approved for clinical application can become excellent models, and scientists continue to improve the carrier or explore the feasibility of other combination agents on the basis of them. In the field of agent research and development, safety is more important than efficacy.

**Figure 1:**
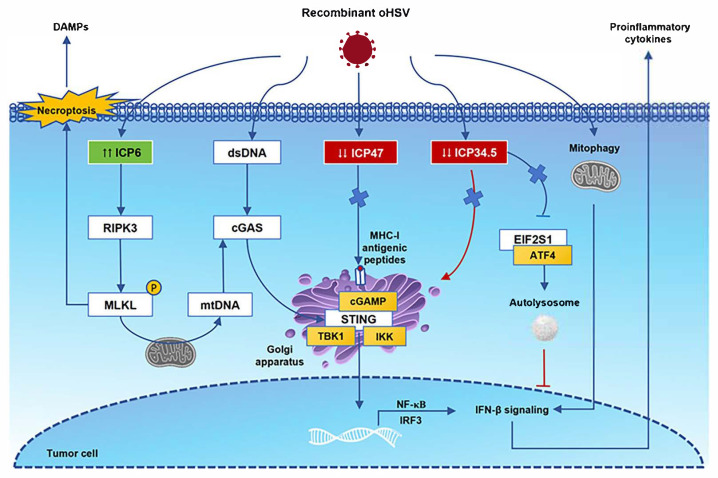
Immune antitumor effects following oncolytic herpes simplex virus (oHSV) infection. After oHSV infection, cyclic GMP-AMP synthase (cGAS) recognizes viral dsDNA, activates STING signaling, recruits TBK1, and promotes the phosphorylation of IRF3. These receptors trigger downstream signaling cascades that lead to NF-κB expression and the release of proinflammatory cytokines. In the cytoplasm, viral proteins ICP47 and ICP34.5 are deleted, which processes MHC-I antigenic peptide presentation and the EIF2S1-ATF4 pathway-associated autolysosome. The ICP6 protein can interact with receptor-interacting kinase-3 (RIPK3) directly. The Rhim domains are necessary for the formation of the ICP6-RIPK3 complex, and they activate RIPK3/mixed lineage kinase domain-like protein (MLKL)-dependent programmed cell necrosis. MLKL-mediated necroptosis will induce cell membrane rupture and release damage-associated molecular patterns (DAMPs) in the TME. Phosphor-MLKL can also translocate to mitochondria, lead to the microtubule-dependent release of mitochondrial DNA (mtDNA), and activate the cGAS-STING signaling pathway. Abbreviations: STING, stimulator of interferon gene; IRF3, interferon regulatory factor 3; TBK1, TANK-binding kinase 1; EIF2S1, eukaryotic translation initiation factor 2 subunit alpha; ATF4, activating transcription factor 4.

HSV-1 has become a commonly used oncolytic viral vector mainly because of its four advantages: a large genome, allowing it to carry ~30 kb of exogenous genes; efficient replication in various tumor cells with a high expression level of the HSV-1 receptor Nectin1; the lysis of tumor cells; and the intrinsic induction of both innate and adaptive antitumor immunity [75]. We used HSV-1 as an example to explore the molecular processes that regulate antitumor immunity after the virus infection of tumor cells and to explore the safety and design principles of its application from the perspective of molecular mechanisms (Figure 1). The safety of oncolytic viruses needs to consider the dual effects of antiviral and antitumor immunity, which requires the vector to have high immunogenicity to activate immunity and to prevent overstimulated immune inflammation. The cyclic GMP-AMP synthase–stimulator of interferon genes (cGAS-STING) pathway is the core pathway mediating cytoplasmic DNA-triggered immune responses. Virus DNA released in the tumor cell cytoplasm is activated and activates cGAS to recognize and synthesize GTP after HSV infection. Subsequently, cGMP-AMP (cGAMP) binds to STING and recruits TANK-binding kinase 1 (TBK1), promoting the phosphorylation of IRF3. Type I IFN transcription and proinflammatory factor expression are induced by interferon regulatory factor 3 (IRF3) and NF-κB [76]. LRRC8A/SWELL1 is a subunit of the volume-regulated anion channel, which is necessary for the transfer of cGAMP into bystander cells [77]. The γ1 34.5 gene of HSV-1 encodes the ICP34.5 protein, which disrupts STING translocation from the endoplasmic reticulum to the Golgi apparatus and compromises the host’s antiviral immunity [78]. Another selective form of autophagy protects mitochondrial homeostasis, regulates the EIF2S1-ATF4 pathway through HSV-1 protein ICP34.5 to block Parkin-mediated mitophagy, and induces NF-κB-mediated neuroinflammation [79]. Therefore, many oncolytic viruses have been designed to delete the γ1 34.5 gene in order to reduce the weakening of antiviral immunity and the probability of neurotoxicity. ICP47 inhibits the stabilization and translocation of major histocompatibility complex class I (MHC-I) antigenic peptides into the Golgi apparatus, which are necessary in CD8^+^ cytotoxic T lymphocyte (CTL) immune surveillance [80]. Although ICP47 binds to the peptide binding site of the transporter associated with antigen presentation, removing ICP47 will benefit oncolytic virus therapy [81]. This may be related to the host’s peptide polymorphism [82]. During the process of viral replication, the ICP6 protein participates in the reduction of ribonucleotides to deoxyribonucleotides, providing raw materials for the synthesis of viral DNA, and is a key link in the DNA replication of viruses within host cells. The ICP6 protein can also interact with receptor-interacting kinase-3 (RIPK3). The Rhim domains of the two proteins are necessary conditions for the formation of the ICP6-RIPK3 complex, which directly activates RIPK3/mixed lineage kinase domain-like protein (MLKL)-dependent programmed cell necrosis and triggers antiviral defense [83,84]. MLKL-mediated necroptosis will induce cell membrane rupture and release damage-associated molecular patterns (DAMPs) in the TME. Phosphor-MLKL can also translocate to mitochondria, lead to the microtubule-dependent release of mitochondrial DNA (mtDNA), and activate the cGAS-STING signaling pathway [85].

**Figure 2:**
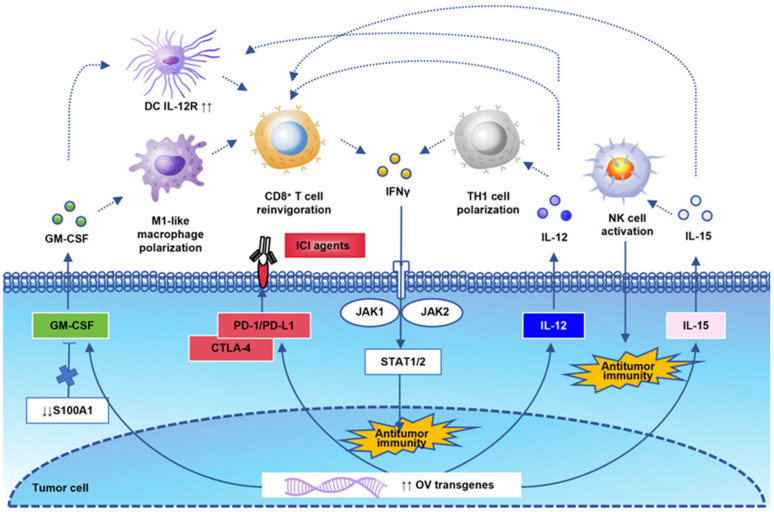
A schematic representation of OV transgenic signaling pathways. The interaction of OV transgenes is the theoretical basis for antitumor immune activation. We draw a schematic diagram of the most classical genes mentioned in this review and their correlation with each immune cell. GM-CSF could activate M1-like macrophage polarization, CD8^+^ T-cell reinvigoration, and upregulate IL-12R on DCs. S100 calcium-binding protein A1 (S100A1) is a key upstream regulator of the GM-CSF axis. IL12 and IL15 are important antitumor immune cytokines, which activate TH1 cells, NK cells, and CD8^+^ T cells, and they play an antitumor immune killing role through the IFNγ signaling pathway. PD-1 and CTLA4 are also commonly used transgenes that enhance the synergistic effect of OV in combination with ICI agents.

In addition to the modification of the HSV genome itself, the design of transgenes and other immunotherapy combinations also needs to be based on the synergy of antitumor immunity. Prior to 2020, GM-CSF was the most common transgene in OV clinical trials [86]. The statistics of 2022 show that the distribution of the top three transgenes has become IL12R, PD-1, and PD-L1 [87]. This reflects the rapid changes in the OV field to a certain extent. In this review, we summarize and analyze the latest research on the mechanisms of these commonly used oncolytic virus transgenes in antitumor immune regulation so as to provide basic theoretical evidence for oncolytic viruses combined with other immunotherapies (Figure 2). GM-CSF was initially regarded as a hematopoietic growth factor, but now, it is a central mediator of T cells and myeloid cells in immunopathology [88]. The modulation of the GM-CSF axis could potentiate an inflamed TME by promoting M1-like macrophage polarization and CD8^+^ T-cell reinvigoration. S100 calcium-binding protein A1 (S100A1) is a key upstream regulator of the S100A1/USP7/p65/GM-CSF axis. The blockade of S100A1 will remodel the inflamed and immunoactive TME [89]. Engineered GM-CSF will potentiate the DC response to IL-12 and enhance the expression of proinflammatory cytokines and chemokines. Subsequently, IL-12 exposure will induce TH1 cell polarization and active IFNγ antitumor immunity [90]. IL-12 will generate interferon-γ (IFNγ) production in both innate and adaptive immune cells [91]. Of course, there are many other oncolytic viral designs targeting different antitumor mechanisms. TG6050, an oncolytic vaccinia virus encoding IL12 and the anti-CTLA-4 antibody, can induce an inflamed TME in metastatic non-small cell lung cancer [92]. PARP1 has been identified as a replication restriction factor of HSV-1 after OV injection in mouse models. The combination of PARPi and an immune checkpoint inhibitor can enhance the ability of OV [93]. As shown in another study, a combination of adoptively transferred T cells or mRNA vaccine with OV therapy can significantly increase both cytokine and CD8^+^ T-cell recruitment [94]. Modulating gut microbiota homeostasis is another supplement combination method used to improve OV therapeutic outcomes via the IL6-JAK-STAT3 signaling pathway [95]. OV has also been used as a replicating vehicle for artificial microRNAs to induce tumor cell killing [96]. There have also been other studies in which chemokine C-C motif ligand 5 (CCL5) and EGFR antibodies have been loaded into oHSV-1, allowing it to recruit immunocompetent cells and inhibit EGFR signaling in the TME of GBM-bearing mice [97]. Researchers have formed a certain consensus that preclinical oncolytic viruses no longer represent a single immune regulatory mechanism but a combination of multiple immune regulatory pathways.

## 4. The Effectiveness and Systemic Delivery Dilemmas of Oncolytic Viruses

From the perspective of indications, current successful clinical cases mainly focus on cancer types such as melanoma, liver cancer, glioblastoma, and bladder cancer. One of the common features of these tumors is that the tumor lesions are easy to locate. Currently, intratumoral injection is still one of the most effective ways to deliver oncolytic viruses in clinical trials. The limitations of the systemic delivery of OVs are mainly affected by the immune clearance of neutralizing antibodies (nAbs), tumor targeting off-targets, and concern about serious side effects [98]. Due to the presence of tumor neovascularization, deep tumors or metastatic tumors often rely more on intravenous agents to achieve tumor targeting. The immune infiltration of the tumor microenvironment is still one of the hotspots in the research of tumor immunotherapy.

Several mechanisms participate in the process of the antibody-mediated neutralization of enveloped viruses, for example, specifically, recognition and binding to viral spike proteins, the aggregation of virions, and blocking virus entry into host cells [99]. Therefore, replacing the capsid protein targeted by neutralizing antibodies is one of the strategies used for oncolytic virus gene editing. A chimeric virus, Delta-24-RGD-H43m, was generated by replacing serotype 5 capsid protein hexon hypervariable regions (HVRs) with serotype 43 to evade neutralizing anti-Ad5 antibodies [100]. Similarly, another study replaced serotype 5 HVR 1 and 5 regions with serotype 35 [101]. However, given that oncolytic viruses often require multiple doses to achieve adequate therapeutic efficacy, it is still possible to generate new neutralizing antibodies against chimeric capsid antigens, although capsid replacement can partially reduce established neutralizing antibodies. Alternatively, physical shielding or competitive binding can obscure the recognition site of neutralizing antibodies and thereby evade clearance by innate immunity. VCN-11 was engineered with an albumin-binding domain on the hexon region of Ad5 and was confirmed to have antitumor efficacy intravenously in the presence of nAbs against Ad5 [102]. SJ-600 is an oncolytic vaccinia virus that expresses the human CD55 protein on the intracellular mature virion membrane. Higher resistance to serum nAbs and complement-mediated lysis has been identified in human xenograft models [103]. Glycosylated polyethylene glycol modified oHSV can escape from nAbs and exhibit high specificity to hepatocellular carcinoma cells while overexpressing the asialoglycoprotein receptor [104]. It is important to note that capsid proteins play a role in the assembly and stability of the virus. Therefore, gene-edited capsid proteins also bring new challenges to the subsequent mass production of stable, standardized agents. There is another new hypothesis that preventing the formation of the virus protein corona, rather than the binding of nAbs or complements, is more important to prolong the circulation time and increase the distribution of OV [105]. Due to the complex mechanism of antitumor immunity, some studies have also confirmed that although nAbs affect the oncolytic effect of viruses, they still have antitumor immunity when combined with other T cell-based immunotherapies such as ICI [106]. Altogether, the modification of the viral capsid for binding to nAbs is effective but not essential. The activation of multiple antitumor immune signaling pathways is more important than the evasion of innate immune recognition.

In addition to simple capsid modification to evade neutralizing antibodies, oncolytic viruses can also load other tumor-specific binding molecules to enhance their tumor targeting. Some novel agents are gradually being tested in advanced solid tumors such as ovarian cancer. One approach is to carry molecules that bind specifically to a particular tumor. For instance, T cell-specific antigens are engineered and expressed on the virus surface to conjugate onto T cells; thus OV will be carried by T cells and infiltrate into the TME to play a role [107]. TILT series viruses are chimeric human 5/3 oncolytic adenoviruses loaded with additional dual regulatory immune molecules along with capsid modification. One product named TILT-123 is a chimeric oncolytic adenovirus armed with TNFα and IL-2, and it has been utilized in early clinical trials (NCT04695327, NCT04217473, and NCT05271318) [108,109,110]. A total of 52 solid tumor patients were treated with TILT-123 intravenously with no dose-limiting toxicities and a prolonged median overall survival [111]. Another product named TILT-322 is armed with a human aMUC1aCD3T cell engager and IL-2, which can stimulate gamma delta T-cell activation and reverse T-cell exhaustion in an ovarian cancer model [112]. An engineered oHSV expressing anti-47 mAbs can block the CD47-SIRPα signaling pathway, enhancing macrophage phagocytosis against ovarian cancer cells [113]. Another approach is to use the properties of biomaterials, such as nanomaterials, to deliver agents into the TME. For example, engineered oHSV with biocompatible magnetic nanoparticles enables the OV targeting of tumors with magnetic guidance [114]. From this research progress, it can be summarized that the development and transformation of oncolytic virus therapy have made a big step forward. Intravenous administration is no longer an absolute barrier to the utilization of oncolytic viruses. With the gradual completion of clinical trials, oncolytic virus research is about to reach new milestones [115].

## 5. Conclusions and Future Prospects

Chronic inflammation and acute inflammation facilitate tumor progression and tumor regression, respectively. Multiple signaling pathways, chemokines, and growth factors are involved in complex dynamic equilibrium processes [116]. Due to the strong plasticity of oncolytic viruses, individualized treatment among different tumor subtypes or different molecular characteristics will be the development trend in oncolytic virus therapy in the future. Therefore, the biomarker development of treatment effectiveness and research on anti-agent resistance mechanisms will be more and more important. No single biomarker can explain the heterogeneous response patterns of OV in pancreatic ductal adenocarcinoma (PDAC) [117]. There are also a few studies on the resistance mechanisms of oncolytic viral agents. One study found the presence of IGF2BP3-induced neutrophil extracellular trap (NET) formation in malignant glioma, and blocking this process by a BET inhibitor can enhance VSVΔ51 replication [118]. IDH1 mutation may lead to impaired type I IFN response and enhance the susceptibility of gliomas to VSVΔ51 infection [119].

On the other hand, the application of oncolytic viruses in the field of neoadjuvant therapy (NAT) also deserves attention. The first study discussing OV as a NAT was published in 2003 [120]. Apart from T-Vec, Pexa-Vec has also been used via intravenous administration before surgery in nine patients with colorectal cancer liver metastases or metastatic melanoma among the representative successful cases we mentioned above [34]. Another oncolytic virus, orienX010, has been assessed in thirty stage III/IV acral melanoma patients in a combination of an anti-PD-1 drug before surgery. Increased radiographic or pathological response rates and higher 2-year RFS/EFS have been detected [121]. Neoadjuvant OV therapy can also be used as an ICI-sensitizing approach in TNBC patients [122]. Up to now, large-sample studies focusing on this field have been rare, and they are often accompanied by the combined use of ICIs or sequential treatment.

In summary, in addition to the issues of safety, effectiveness, and administration routes that we focused on in this article, there are also some technical barriers in the production and purification process. Innovative vector designs and recombinant genes will gradually be improved and simplified based on successful cases. True clinical applications hope to achieve safe and precise treatment. Due to the high heterogeneity of the tumor microenvironment, the field of OV treatment in the future will be a colorful scene. In the future, oncolytic virus therapy will not be monopolized by star products like T-VEC, but rather, diverse and precise treatment should be achieved, targeting different tumor signaling pathways or immune regulatory mechanisms.

## Figures and Tables

**Table 1:** Overview of typical recombinant oncolytic virus agents in chronological order.

Agent	Virus	Construction Scheme	Indication	Route of Administration *
H101	Adenovirus	Deleted E1B-55kD	HNSCC	*
T-VEC	HSV-1	Expressing GM-CSF	Melanoma	*
JX-594	Poxvirus	Deleted TK, expressing GM-CSF	HCC	Intravenous injection
G47Δ	HSV-1	Deleted ICP34.5 and ICP47, inactivated ICP6	GBM	*
RP1	HSV-1	Expressing GALV-GP-R- and GM-CSF	Melanoma	*
CG0070	Adenovirus	Deleted E1A, expressing GM-CSF	NMIBC	Intravesical injection
OH2	HSV-2	Deleted ICP34.5 and ICP47, expressing GM-CSF	Melanoma	*
PVSRIPO	Polio–rhinovirus chimera	Polio–rhinovirus chimera, expressing CD155	Melanoma and GBM	Intratumoral and intrathecal injection
Olvi-Vec	Vaccinia virus	Replacing the TK, hemagglutinin, and F145L genes with three expression cassettes encoding β-galactosidase, β-glucuronidase, and RLuc-GFP fusion proteins	OV and lung cancer	Intraperitoneal injection and intrathoracic injection
Reolysin	Reovirus	A type 3 oncolytic reovirus hijacking the Ras signaling pathway	Breast cancer and MPA	Intravenous injection
VG161	HSV-1	Deleted ICP34.5, expressing IL-12, IL-15, IL-15Rα, and PD-1/PD-L1	HCC	*
NDV-GT	Newcastle disease virus	Expressing porcine α1,3 GT gene	HCC	Intravenous injection

* All agents without the mode of administration indicated are given through intratumoral injection. Abbreviations: HNSCC, head and neck squamous cell carcinoma; GM-CSF, granulocyte–macrophage colony-stimulating factor; HSV-1, herpes simplex virus type 1; TK, thymidine kinase; HCC, hepatocellular carcinoma; GBM, glioblastoma; NMIBC, non-muscle-invasive bladder cancer; OV: ovarian cancer; MPA: metastatic pancreatic adenocarcinoma.

**Table 2:** Completed phase 2/3 clinical trials of typical oncolytic virus agents.

Agents	Indications	NCT Number	Enrollment	Phase	Arms	ORR	OS	TRAEs *	PMID
H101	Head and neck or esophagus squamous cell cancer	-	160	3	A: H101 + PF/AF B: PF or AF	H101 + PF = 78.8% PF = 39.6% H101 + AF = 50.0% AF = 50.0%	Unknown	None	15601557
H101	Refractory malignant ascites	NCT04771676	25	2	H101	Unknown	Increased median time to repeat paracentesis of 45 days from 13 days.	8.0%	38659226
T-VEC	Unresected stage IIIB to IV melanoma	NCT00769704	436	3	A: T-VEC B: GM-CSF	The ORR was 26.4% in arm A.	The median OS was 23.3 months with T-VEC and 18.9 months with GM-CSF.	A: 13.4% B: 7.1%	26014293
T-VEC	Unresected stage IIIB to IVM1c melanoma	NCT02263508 (Terminated)	692	3	A: T-VEC + Pembrolizumab B: Placebo + Pembrolizumab	The ORR was 48.6% in arm A and 41.3% in arm B.	T-VEC-pembrolizumab did not significantly improve PFS or OS compared with arm B.	A: 20.7% B: 19.5%	35998300
T-VEC	Unresected stage IIIB to IVM1c melanoma	NCT02211131	150	2	A: Neoadjuvant T-VEC + surgery B: Surgery alone	Pathological CR was 17.1% in arm A.	The 2-year OS was 88.9% for arm A and 77.4% for arm B.	5.5%	34608333
T-VEC	Unresected stage IIIB to IV melanoma	NCT01740297	198	2	A: T-VEC + Ipilimumab B: Ipilimumab alone	The ORR was improved from 16.0% in arm B to 35.7% in arm A.	The estimated 5-year OS increased from 48.4% in arm B to 54.7% in arm A.	A: 46.3% B: 43.2%	37142291
JX-594	Advanced hepatocellular carcinoma	NCT01721772 (Terminated)	459	3	A: JX-594 + Sorafenib B: Sorafenib alone	The ORR was 19.2% in arm A and 20.9% in arm B.	The median OS was 12.7 months in arm A and 14.0 months in arm B.	A: 53.7% B: 35.5%	38756145
RP1	Anti-PD-1-failed melanoma	NCT03767348	140	2	RP1 + Nivolumab	The ORR was 32.9%	Overall survival rates at 1 and 2 years were 75.3% and 63.3%, respectively.	12.9%	40627813
CG0070	BCG-unresponsive non-muscle-invasive bladder cancer	NCT02365818	45	2	CG0070	The overall 6-month CR was 47.0%.	Unknown	4.5%	28755959
PVSRIPO	Recurrent supratentorial glioblastoma	NCT04479241	25	2	PVSRIPO + Pembrolizumab	Unknown	The median OS was 10.2 months.	32.0%	Unpublished
PVSRIPO	Recurrent WHO grade IV malignant glioma	NCT02986178	121	2	A: PVSRIPO B: PVSRIPO + Lomustine	The ORR was 5.4% in arm A and 7.4% in arm B.	The median 5-year OS was 7.0 months in arm A and 7.1 months in arm B.	A: 18.1% B: 30.8%	Unpublished
Olvi-Vec	Platinum-resistant or platinum-refractory ovarian cancer	NCT02759588	27	2	Olvi-Vec + platinum-based chemotherapy with or without bevacizumab	The ORR was 54.0%.	The median OS was 15.7 months in all patients.	11.1%	37227734
Reolysin	Hormone receptor+, HER2-metastatic breast cancer	NCT04215146	48	2	A: Paclitaxel B: Paclitaxel + Reolysin C: Paclitaxel + Reolysin + avelumab	The 16-week ORR was 20.0% (arm A), 31.0% (arm B), and 14.0% (arm C).	The median PFS was 6.4 (arm A), 12.1 (arm B), and 5.8 months (arm C).	A: 16.7% B: 87.5% C: 148.0%	40300087

**Abbreviation:** ORR: overall response rate; PFS: progression-free survival; RFS: recurrence-free survival; OS: overall survival; TRAEs: treatment-related adverse events; PF: cisplatin plus 5-fluorouracil; AF: Adriamycin plus 5-fluorouracil; BCG: bacillus Calmette–Guérin; CR: complete response. * The TRAEs mentioned here refer to the percentage of adverse events ≥ grade 3.

**Table 3:** Ongoing phase 3 clinical trials of oncolytic virus agents among diverse cancer types.

Indications	Trial Identifier	Agent	Estimated Enrollment	Arms	Estimated Study Completion Date
Patients with unresectable or metastatic melanoma who have failed at least second-line standard therapy	NCT05868707	OH2	340	A: OH2 B: Salvage chemotherapy or best supportive care	March 2027
Patients with unresectable stage IIIb-IV cutaneous melanoma whose disease progressed on an anti-PD-1- and anti-CTLA-4-containing regimen or who are not candidates for treatment with an anti-CTLA-4 therapy	NCT06264180	RP1	400	A: RP1 + Nivolumab B: Nivolumab + Relatlimab (as Opdualag) C: Anti-PD-1 monotherapy (nivolumab or pembrolizumab) D: Single-agent chemotherapy (dacarbazine, temozolomide, or paclitaxel/albumin-bound paclitaxel)	August 2034
Intermediate risk non-muscle-invasive bladder cancer following transurethral resection of bladder tumor	NCT06111235	CG0070	364	A: CG0070 B: Surveillance	January 2030
Platinum-resistant/refractory ovarian cancer, fallopian tube cancer, and primary peritoneal cancer	NCT05281471	Olvi-Vec	186	A: Olvi-Vec + Platinum-doublet and bevacizumab B: Chemotherapy and bevacizumab	October 2026

## Data Availability

No new data were created or analyzed in this study.

## Abbreviations

Table following abbreviations are used in this manuscript:

AE	Adverse event
ATF4	Activating transcription factor 4
CDC	Complement-dependent cytotoxicity
cGAS-STING	Cyclic GMP-AMP synthase–stimulator of interferon genes
CLEC10A	C-type lectin domain-containing 10A
CRC	Colorectal cancer
CTL	Cytotoxic T lymphocyte
DAMPs	Damage-associated molecular patterns
DC	Dendritic cell
EGFR	Epidermal growth factor receptor
EIF2S1	Eukaryotic translation initiation factor 2 subunit alpha
GM-CSF	Granulocyte–macrophage colony-stimulating factor
GZMK	Granzyme K
HCC	Hepatocellular carcinoma
HSV	Herpes simplex virus
HVR	Hexon hypervariable region
ICI	Immune checkpoint inhibitor
IFNγ	Interferon-γ
IL-12	Interleukin-12
IRF3	Interferon regulatory factor 3
MDSC	Myeloid-derived suppressor cell
MHC-I	Major histocompatibility complex class I
MPA	Metastatic pancreatic cancer
NAb	Neutralizing antibody
NK cells	Natural killer cells
NMIBC	Non-muscle-invasive bladder cancer
ORR	Objective response rate
OV	Oncolytic virus
PDAC	Pancreatic ductal adenocarcinoma
S100A1	S100 calcium-binding protein A1
STING	Stimulator of interferon gene
TAM	Tumor-associated macrophage
TAP	Transporter-associated peptide
TBK1	TANK-binding kinase 1
TIME	Tumor immune microenvironment
Treg cell	Regulatory T cell
TME	Tumor microenvironment
